# Model‐Based Assessment of Lecanemab Maintenance Dosing Regimen Shows Continued Suppression of Amyloid Plaque, Disease Progression

**DOI:** 10.1002/alz.089308

**Published:** 2025-01-09

**Authors:** Brian A. Willis, Pratik Bhagunde, Robert Bell, Natasha Penner, Arnaud Charil, Larisa Reyderman

**Affiliations:** ^1^ Eisai Inc., Nutley, NJ USA

## Abstract

**Background:**

Lecanemab is a humanized IgG1 monoclonal antibody that binds with high affinity to Aβ soluble protofibrils. In two clinical studies (phase 2, NCT01767311 and phase 3 ClarityAD, NCT03887455) in early Alzheimer’s disease, lecanemab substantially reduced amyloid PET and significantly slowed clinical decline on multiple measures of cognition and function, including CDR‐SB at 18 months. Models describing the change in amyloid PET and CDR‐SB in response to lecanemab treatment were used to explore the impact of changing from the initial dosage regimen (10 mg/kg every 2 weeks [Q2W]) to a less intensive maintenance dosing regimen (10 mg/kg every 4 weeks [Q4W]) on clinical efficacy, and to explore the optimal duration of the initial dosing regimen.

**Methods:**

Models describing the relationship between serum lecanemab exposure, amyloid PET, and CDR‐SB have been previously described. Individual post hoc model estimates for pharmacokinetics, PET, and CDR parameters were obtained from Clarity AD subjects who received lecanemab and were used to simulate change in amyloid PET and CDR‐SB over 4 years. Simulations were conducted to evaluate the effect of lecanemab after continuous Q2W treatment or transitioning to a less intensive dosing regimen (10 mg/kg Q4W) at 18 or 24 months. Simulations were also evaluated for the subgroups of subjects with baseline amyloid PET <60 CL and ≥60 CL.

**Results:**

After 18 months of lecanemab treatment, the mean amyloid PET level was ≤25 CL in both the high and low baseline PET groups. Continued lecanemab treatment with Q2W and Q4W resulted in a similar additional reduction of amyloid PET over 4 years of treatment. The difference in amyloid reduction between continuous Q2W treatment and Q4W treatment initiated at 18 months was 2.5 CL and 5.1 CL in the low and high baseline PET groups respectively. No significant difference in CDR‐SB was projected between the two groups, regardless of when the subjects transitioned.

**Conclusions:**

Subjects receiving lecanemab 10 mg/kg Q2W may transition to a less intensive (Q4W) maintenance regimen after 18 months. The maintenance regimen has no clinically meaningful impact on amyloid or disease progression relative to the Q2W regimen.

